# Oral N-Acetyl-Cysteine Attenuates Loss of Dopaminergic Terminals in α-Synuclein Overexpressing Mice

**DOI:** 10.1371/journal.pone.0012333

**Published:** 2010-08-23

**Authors:** Joanne Clark, Elizabeth L. Clore, Kangni Zheng, Anthony Adame, Eliezer Masliah, David K. Simon

**Affiliations:** 1 Department of Neurology, Beth Israel Deaconess Medical Center, Boston, Massachusetts, United States of America; 2 Harvard Medical School, Boston, Massachusetts, United States of America; 3 Department of Neuroscience, School of Medicine, University of Southern California, San Diego, California, United States of America; National Institutes of Health, United States of America

## Abstract

Levels of glutathione are lower in the substantia nigra (SN) early in Parkinson's disease (PD) and this may contribute to mitochondrial dysfunction and oxidative stress. Oxidative stress may increase the accumulation of toxic forms of α-synuclein (SNCA). We hypothesized that supplementation with n-acetylcysteine (NAC), a source of cysteine – the limiting amino acid in glutathione synthesis, would protect against α-synuclein toxicity. Transgenic mice overexpressing wild-type human α-synuclein drank water supplemented with NAC or control water supplemented with alanine from ages 6 weeks to 1 year. NAC increased SN levels of glutathione within 5–7 weeks of treatment; however, this increase was not sustained at 1 year. Despite the transient nature of the impact of NAC on brain glutathione, the loss of dopaminergic terminals at 1 year associated with SNCA overexpression was significantly attenuated by NAC supplementation, as measured by immunoreactivity for tyrosine hydroxylase in the striatum (p = 0.007; unpaired, two-tailed t-test), with a similar but nonsignificant trend for dopamine transporter (DAT) immunoreactivity. NAC significantly decreased the levels of human SNCA in the brains of PDGFb-SNCA transgenic mice compared to alanine treated transgenics. This was associated with a decrease in nuclear NFκB localization and an increase in cytoplasmic localization of NFκB in the NAC-treated transgenics. Overall, these results indicate that oral NAC supplementation decreases SNCA levels in brain and partially protects against loss of dopaminergic terminals associated with overexpression of α-synuclein in this model.

## Introduction

Parkinson's disease (PD) is a progressive neurodegenerative disorder involving loss of specific subsets of neurons, including dopaminergic neurons of the substantia nigra (SN). Though the precise causes of PD are unknown, a large body of evidence implicates mitochondrial dysfunction and oxidative stress [1], [2]. Mitochondrial complex I function is impaired early in the course of PD [3], and pharmacological inhibitors of complex I lead to some of the features of PD in animal models [4], [5], suggesting that mitochondrial complex I deficiency may play a role in the pathogenesis of PD. Complex I impairment leads to an increase in the generation of reactive oxygen species (oxidative stress) [6]–[8], consistent with reports of elevated markers of oxidative damage to lipids, proteins, and DNA in the SN in PD [9]. This problem is compounded by the fact that levels of glutathione, the predominant intracellular thiol antioxidant, are severely deficient in the SN at very early stages of PD [10], [11].

SNCA toxicity also plays a central role in PD [12]. Although the mechanisms of this toxicity are unknown, SNCA enhances susceptibility to oxidative stress in a dopamine-dependent manner [13]. This susceptibility may be due to the increased tendency of SNCA to aggregate when exposed to oxidative stress [14]–[18], and the stabilization of a toxic protofibril form of SNCA by oxidative ligation of SNCA to dopamine [19]. If correct, then increased oxidative stress due to early glutathione deficiency in the SN may lead to enhanced toxicity of SNCA in dopaminergic SN neurons, suggesting that strategies to increase glutathione or to block oxidative stress by other means may protect against SNCA toxicity.

We tested this hypothesis in an animal model of PD. Mice overexpressing wild-type human SNCA from the platelet-derived growth factor beta (PDGFb) promoter (line D PDGFb-SNCA) are reported to develop motor impairments in association with progressive loss of dopaminergic terminals [20]. Autosomal dominant PD, which is clinically similar to idiopathic disease, can be caused by a duplication or triplication of the normal SNCA gene resulting in a global increase in brain SNCA expression [21]–[23], demonstrating the relevance of this mouse model to human disease. The impact of oral NAC supplementation from weaning until 1 year of age was determined in these mice. NAC can raise glutathione levels by acting as a cysteine donor in the synthesis of glutathione [24]. NAC also has direct antioxidant activity and additional effects on various cellular kinases and transcription factors, including NFκB [25]. Systemic administration of NAC increases brain levels of glutathione in mice [26]–[30], reduces markers of oxidative damage [29], increases brain synaptic mitochondrial complex I activity [28], and protects against MPTP toxicity [31]–[33]. Oral NAC is well tolerated even in elderly humans [34] and has been proposed as a possible neuroprotective agent in PD [35]–[37], but data in a chronic degenerative animal model of PD has been lacking. The aim of this work was to test our hypothesis that oral NAC supplementation could be neuroprotective in a chronic model of SNCA overexpression. The results support this hypothesis, as striatal TH+ terminal density was increased in NAC-treated SNCA overexpressing mice compared to SNCA overexpressing mice on a control diet and this correlated with a decrease in SNCA immunoreactivity in the brains of SNCA overexpressing mice treated with NAC.

## Results

### Chronic oral treatment with NAC increases the density of TH+ terminals in SNCA-overexpressing mice

**Figure 1:**
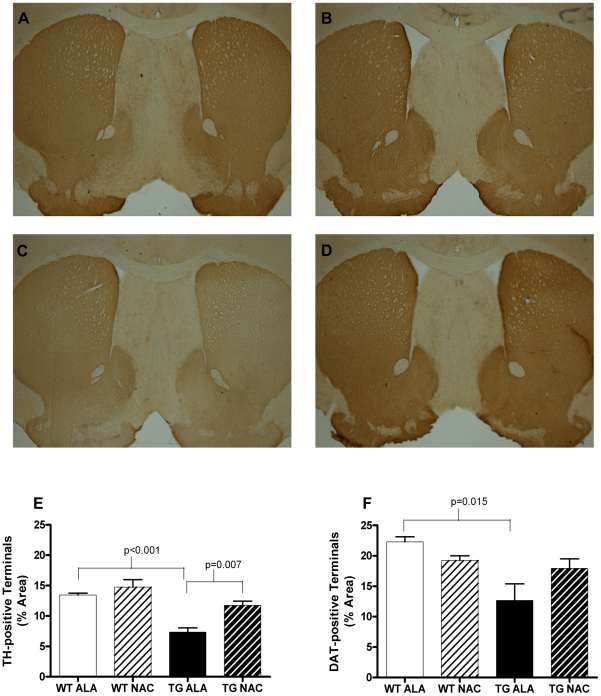
NAC increases striatal area occupied by TH-positive terminals in SNCA-overexpressing mice at 12 months of age. A–D. Representative images of TH-immunostained 30 µm sections of alanine or NAC-treated wild-type or transgenic mouse striatum. A. Wild-type, alanine treated; B. Wild-type, NAC treated; C. Transgenic, alanine treated; D. Transgenic, NAC treated. E. Density of TH-positive terminals in the striatum of alanine or NAC-treated wild-type or transgenic mouse striatum. F. Density of DAT-positive terminals in the striatum of alanine or NAC-treated wild-type or transgenic mouse striatum. Data in E and F were analyzed using a 2-tailed Student's t-test. All relevant statistically significant comparisons are indicated on the graph. WT ALA N = 4, WT NAC  = 6, TG ALA  = 4, TG NAC  = 3.

Transgenic mice overexpressing SNCA showed a mean decrease of 45.2% (95% CI 31.5–59.0%; p<0.001) in the percentage of striatal area covered by tyrosine hydroxylase positive (TH+) terminals compared to wild-type littermate controls at 1 year of age (**Fig. 1A, 1C,1E**). This loss of TH+ terminals was significantly attenuated by chronic oral NAC supplementation beginning at 3 weeks of age (p = 0.007; **Fig. 1D, 1E**). NAC supplementation did not significantly alter the percentage of striatal area covered by TH+ terminals in wild-type mice. As an additional measure of dopaminergic terminals in the striatum, the percentage of striatal area covered by dopamine transporter positive (DAT+) terminals also was assessed (**Fig. 1F**). Similar to the findings for TH+ terminals, the percentage of striatal area covered DAT+ terminals was reduced by 43.2% (95% CI 12.0–74.3%; p = 0.015) in SNCA overexpressing mice compared to littermate controls (**Fig. 1F**). There was a non-significant trend towards protection against this loss of striatal DAT+ terminals by chronic oral NAC supplementation (p = 0.19).

### Treatment with NAC Reduces Levels of SNCA in PDGFb-SNCA Transgenic Mice

**Figure 2:**
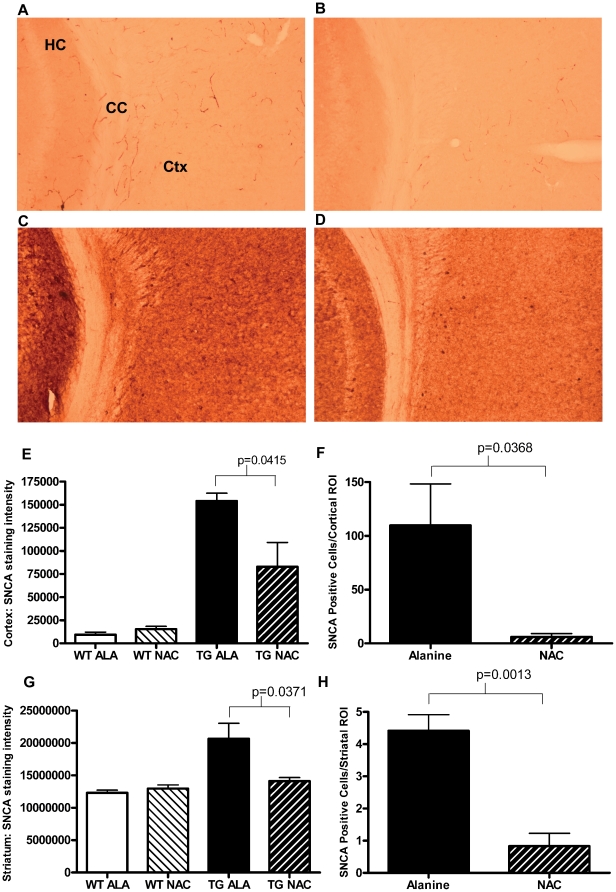
Treatment with NAC Reduces Levels of SNCA in PDGFb-SNCA Transgenic Mice. A–D. Representative images of human SNCA-immunostained 30 µm sections of alanine or NAC treated wild-type or transgenic mouse cortex. A. Wild-type, alanine treated; B. Wild-type, NAC treated; C. Transgenic, alanine treated; D. Transgenic, NAC treated. E. Human SNCA staining intensity (integrated density) of the cortex of wild-type and transgenic mice treated with either alanine or NAC normalized to intensity of staining of the corpus callosum on the same brain section. WT data is shown to demonstrate the level of non-specific background staining. F. Quantification of the number of intensely stained cells per cortical region of interest. G. Human SNCA staining intensity (integrated density) of the striatum of wild-type and transgenic mice treated with either alanine or NAC normalized to intensity of staining data from the corpus callosum of the same brain section. H. Quantification of the average number of intensely stained cells per striatal region of interest. Because of low numbers of SNCA-positive cells in the striatum, ROI data from three separate striatal sections were averaged. Data in E–H were analyzed using a 2-tailed Student's t-test. All relevant statistically significant comparisons are indicated on the graph. All groups N = 4.

Mice treated with NAC exhibited an average 46.2% decrease (95% CI 2.4–89.9%; p = 0.0415) in human SNCA immunoreactivity in the cortex (**Fig. 2A–2E**) compared to PDGFb-SNCA transgenic mice treated with alanine. In addition, PDGFb-SNCA transgenic mice treated with NAC also exhibited 94.5% fewer cells containing SNCA-positive intracytoplasmic inclusions in the cortex (**Fig. 2F**; p = 0.0368), from an average of 109 cells/ROI (SEM±38.64) in PDGFb-SNCA transgenic mice supplemented with alanine, to an average of 6 cells/ROI (SEM±3.24) in PDGFb-SNCA transgenic mice supplemented with NAC.

Similar results were also observed in the striata (**Fig. 2G**) of PDGFb-SNCA transgenic mice treated with NAC, where a 31.6% decrease (95% CI 2.6–60.5% p = 0.0371) in human SNCA immunoreactivity compared to alanine treated animals was observed. NAC treated PDGFb-SNCA transgenic mice also exhibited 81.1% fewer cells containing SNCA-positive intracytoplasmic inclusions in the striatum (**Fig. 2H**; p = 0.0013), from an average of 4.42 cells/ROI (SEM±0.4977) in PDGFb-SNCA transgenic mice supplemented with alanine, to an average of 0.83 cells/ROI (SEM±0.6365) in PDGFb-SNCA transgenic mice supplemented with NAC.

### NAC Treatment Increases Brain Glutathione Levels in the Short-Term

**Figure 3:**
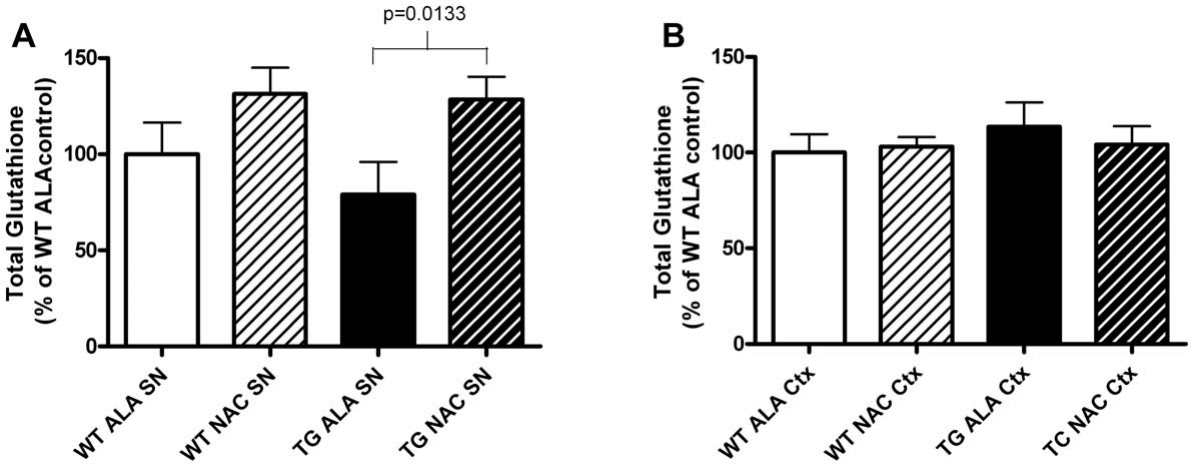
SNCA-Overexpressing Mice Treated with NAC for 5–7 Weeks Exhibit Increased Glutathione in the Substantia Nigra but not in Cortex. A. Levels of total glutathione in the Substantia substantia nigra (SN) B. Levels of total glutathione in the cortex (Ctx) of SNCA overexpressing mice (TG) versus wild-type littermate controls (WT) exposed to drinking water supplemented with NAC or control drinking water supplemented with alanine (ALA). Data were analyzed using a 2-tailed Student's t-test. All relevant statistically significant comparisons are indicated on the graph. WT ALA N = 6, WT NAC N = 6, TG ALA N = 5, TG NAC N = 6.

**Figure 4:**
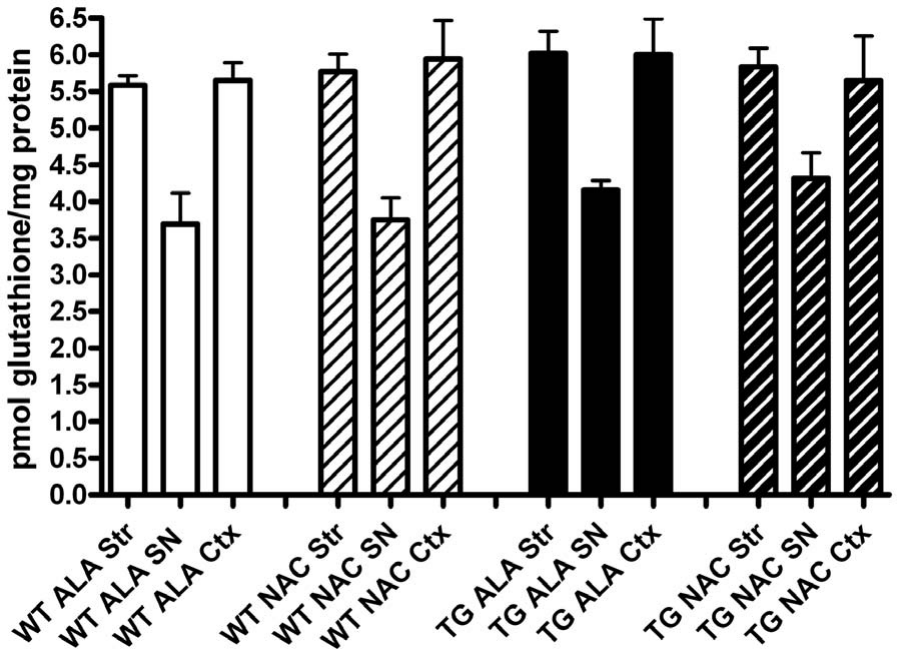
Early increases in SN glutathione after NAC treatment are not seen after 12 months of treatment. Levels of total glutathione in the striatum (Str), substantia nigra (SN) and frontal cortex (Ctx) of SNCA overexpressing mice (TG) versus wild-type littermate controls (WT) exposed to drinking water supplemented with NAC or control drinking water supplemented with alanine (ALA) from weaning through 1 year of age. Though levels of glutathione are consistently lower in the SN compared to Str or Ctx at 1 year of age, there are no significant differences between WT versus TG mice, or between ALA versus NAC treated mice. WT ALA N = 4, WT NAC N = 5, TG ALA N = 3, TG NAC N = 3.

To verify prior studies demonstrating that oral NAC supplementation increases brain levels of glutathione in mice [38], we determined glutathione levels in the SN and cortex of mice with ad lib access to drinking water supplemented with 40 mM NAC for 5–7 weeks, beginning at 3 weeks of age. Control drinking water was supplemented with 40 mM alanine. NAC supplementation significantly increased SN levels of glutathione by a mean of 49% (p = 0.0133) in transgenic mice overexpressing SNCA (**Fig. 3**). However, this initial increase in SN glutathione levels was not seen in older mice that had been supplemented with NAC from weaning until 12-months of age (**Fig. 4**). NAC had no effect on glutathione levels in the cortex at either time-point (**Fig. 3** **&** **4**).

### Treatment with NAC Increases Cytosolic localization of NFκB

**Figure 5:**
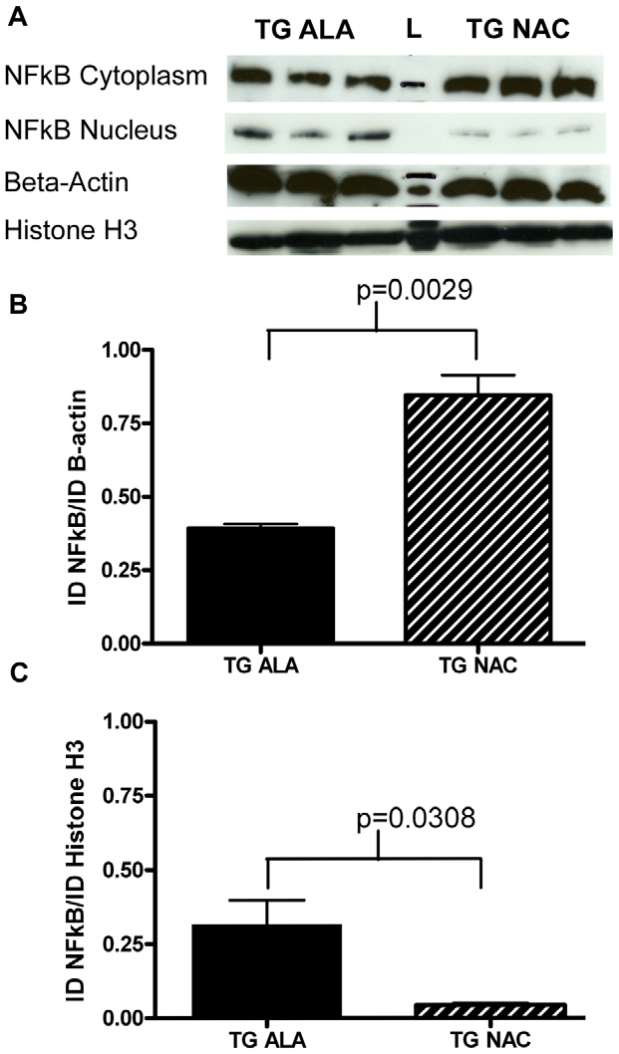
Long-term NAC treatment affects sub-cellular localization of NFκB. A. 2.5 µg of cytoplasmic protein lysate from the cortex of 3 alanine- and 3 NAC-treated PDGFb-SNCA mice was run on a 10-well 4–15% SDS-PAGE gel (Bio-Rad). After transfer, the membrane was cut around the 50 kD marker and probed with anti- NFκB p65 antibody (Santa Cruz) and anti-β-actin antibody (Santa Cruz). 2.5 µg of nuclear protein lysate was run on a separate 10-well 4–15% SDS-PAGE gel and the treated in the manner outlined above. In both cases a single 65 kDa band was observed for NFκB and a single 43 kDa band was observed for β-actin. B. Band quantification of cytoplasmic NFκB normalized to β-actin levels. C. Band quantification of nuclear NFκB normalized to Histone H3 levels. Data were analyzed using a 2-tailed Student's t-test. All relevant statistically significant comparisons are indicated on the graphs. The experiment was repeated three times; representative results from a 1 minute exposure of both blots are shown.

NAC can influence the activity of several transcription factors, including inhibition of NFκB [25], increased activation of NFκB has been observed in Parkinson's disease models [39]–[41], and inhibition of NFκB in glial cells has been proposed as a promising neuroprotective strategy [42]. Nuclear and cytoplasmic fractions were isolated from cortical tissues of wild-type and PDGFb-SNCA mice. Proteins were separated by SDS-PAGE and probed with anti- NFκB p65 antibody (Santa Cruz). An increased amount of NFκB was seen in the cytoplasm of NAC-treated PDGFb-SNCA cortical tissue compared to alanine-treated transgenics (**Fig. 5A & 5B**); accordingly, the amount of nuclear NFκB was decreased in these animals (**Fig. 5A & 5C**). Total NFκB normalized to nuclear and cytoplasmic loading controls did not vary significantly between PDGFb-SNCA transgenic mice treated with either alanine or NAC (data not shown).

NFκB is recruited to the PDGFb promoter in other tissues [43], [44], although this has not been demonstrated in brain. Nevertheless, since SNCA is expressed from the PDGFb promoter in this transgenic model we felt it was necessary to determine whether NAC treatment may be affecting expression of transgenic α-synuclein from the PDGFb promoter. Levels of PDGFb were not significantly different between PDGFb-SNCA mice treated with either alanine or NAC as determined by western blot (see **Supplementary Figure S1**). Therefore the lowering of SNCA protein levels seen in the PDGFb-SNCA transgenic mice after treatment with NAC for one year is unlikely to be due to a downregulation of the PDGFb promoter by NAC.

### Effects of NAC Supplementation on Tests of Motor Coordination

**Figure 6:**
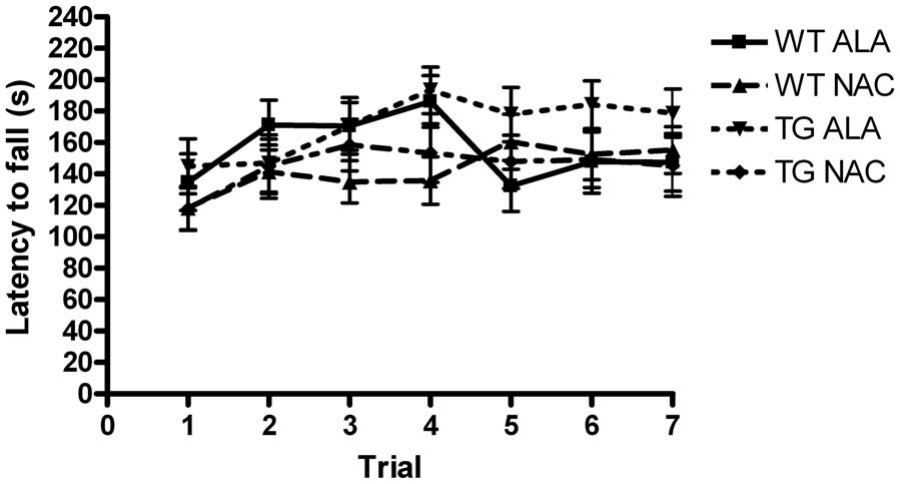
Performance on the accelerating Rotarod was unaffected by SNCA-overexpression or by chronic NAC treatment. Wild-type (WT) and SNCA-overexpressing (TG) mice treated with either Alanine (ALA) or NAC were tested at 1 year of age on a Rotarod (Ugo Basile) accelerating from 2–40 rpm over a period of 240 seconds. There were no significant differences between the groups tested. WT ALA N = 13, WT NAC N = 14, TG ALA N = 12, TG NAC N = 16.

Each cohort of mice was tested on a battery of motor coordination tests at 3, 6 and 12 months of age, including rotarod testing, the pole test, and nest construction. We detected no impairments of motor coordination in any of the groups tested at any of the time points tested. Only data from the rotarod (and pole test) at 12 months of age is shown (**Fig. 6** **&** **7**) and is presented in a style to allow for comparison with previously published work [20].

**Figure 7:**
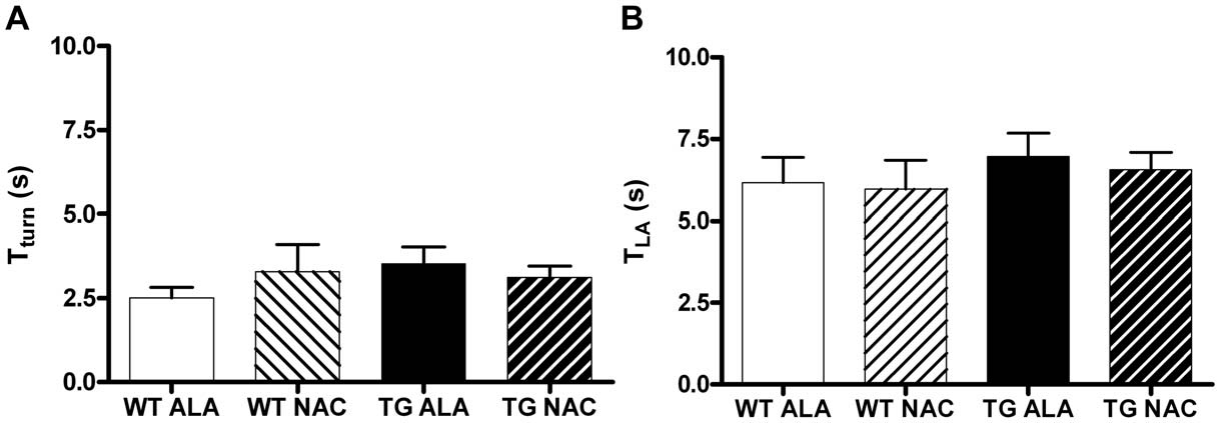
Performance on the Pole Test was unaffected by SNCA-overexpression or NAC treatment. A. Time for the mouse to turn on the pole and face downwards (T_turn_). B. Time for the mouse to reach the bottom of the pole (T_LA_). There were no significant differences between the groups tested. WT ALA N = 12, WT NAC N-12, TG ALA N = 13, TG NAC N = 16.

## Discussion

In this study, we sought to assess the potential for neuroprotection by NAC in a chronic, transgenic mouse model of PD. Multiple transgenic mouse models have been developed based on overexpression of SNCA, each with advantages and limitations [45], [46]. We selected this model (line D PDGFb-SNCA) which overexpresses wild-type human SNCA from the PDGFb promoter, in part, based on the fact that increased expression of wild-type SNCA in humans can cause PD, as demonstrated in families with autosomal dominant PD due to duplication or triplication of the normal (wild-type) SNCA gene [21], [22], [47], [48]. In addition, the line D PDGFb-SNCA mouse model has been reported to show progressive loss of TH+ dopaminergic terminals in the striatum and deficits in rotarod motor performance [20].

As previously reported, we find that mice overexpressing SNCA display a significant loss of striatal TH+ terminals, and now report the novel finding that chronic oral NAC supplementation protects against this loss (**Fig. 1E**). A similar but non-significant trend was seen for terminals labeled with anti-DAT antibody (**Fig. 1F**). It is possible that upregulation of DAT expression in the remaining DA terminals of SNCA overexpressing mice might mask the loss of dopaminergic terminals by this measure, though it also is possible that a difference would have been revealed with a larger number of mice in each group. However, it is worth noting that the protection by NAC in SNCA overexpressing mice against loss of TH+ terminals remains significant even after a conservative Bonferroni correction for multiple comparisons (3 measures of striatal dopaminergic innervation: percentage of area covered by TH+ terminals, percentage of area covered by DAT+ terminals, and striatal dopamine).

NAC significantly reduced the amount of overexpressed human SNCA protein in the cortex and striatum of PDGFb-SNCA transgenic mice (**Fig. 2E–2H**). The mechanism of this effect is yet to be elucidated. NAC can exert antioxidant effects directly by acting as a reducing agent and indirectly by increasing glutathione synthesis [25]. Glutathione levels in the SN were increased after 5–7 weeks of NAC supplementation but this increase was not seen at 1 year (**Fig. 3** **&** **4**). Glutathione levels in the cortex were unchanged by NAC treatment at either time-point. The dopaminergic SN is a region of high oxidative stress compared to the non-dopaminergic cortex [49]. SN GSH levels were higher in the NAC treated groups for both wild type and PDGFb-SNCA transgenic mice, but this increase only reached significance in the case of PDGFb-SNCA mice. The high levels of oxidative stress in the SN may be compounded by the presence of increased SNCA in the PDGFb-SNCA transgenics, and this increased oxidative stress may “prime” the cells of the SN in the SNCA overexpressing mice to upregulate glutathione synthesis when provided with additional cysteine precursor, whereas this may be less necessary in cortical cells.

Since glutathione levels were not altered by NAC in the cortex, it is likely that the observed reduction in anti-SNCA immunoreactivity in the cortex of PDGFb-SNCA transgenic mice after treatment with NAC is independent of any effects of NAC on glutathione synthesis. Levels of SNCA protein have been found to increase upon exposure to oxidative stress [50], potentially through stabilization of SNCA by oxidative ligation to dopamine [19]. Therefore it is feasible that direct antioxidant effects of NAC may have reduced levels of SNCA protein. However, since glutathione was transiently increased in the SN of NAC treated PDGFb-SNCA mice after 5–7 weeks of supplementation (but not after 1 year), we cannot completely exclude the possibility that even a transient rise in glutathione may have had lasting effects.

Long-term treatment with NAC affected NFκB signaling in the brain by increasing cytoplasmic retention of NFκB (**Fig. 5**) thus preventing its action as a transcription factor, which requires translocation to the nucleus. Increased activation of NFκB may contribute to the pathology in models of Parkinson's disease [39]–[41], [51]; therefore, it is possible that protection of striatal TH+ fibers by NAC treatment may be linked to reduced NFκB activity in these animals. Recently published work in the PDGFb-SNCA transgenic line [52] has demonstrated that SNCA is transmitted to astrocytes from neurons, and that the accumulation of SNCA in astrocytes leads to an increased microglial response. Other *in vivo* work also demonstrates that the selective expression of SNCA (in this case the A53T mutant) in astrocytes leads to increased inflammatory responses and microglial activation resulting in significant dopaminergic cell loss [53]. In addition, NFκB inhibitory peptides have been shown to inhibit MPP+ induced activation of NFκB in astrocytes and microglia, which diminished the effects of inflammation and protected the nigrostriatum against MPTP-induced toxicity [51]. Therefore, the reduction of SNCA expression and inhibition of NFκB in glial cells may be a valid strategy for neuroprotection in PD. In this work, we used gross dissections containing a mixed population of both neurons and glia to determine the cellular compartmentalization of NFκB; further studies to isolate the glial population in order to determine the glial cell SNCA load and level of NFκB activation in response to NAC may provide valuable insight into the mechanism of NAC-mediated neuroprotection in this model.

A limitation of the current study is that we were unable to confirm behavioral deficits associated with SNCA overexpression in this mouse model (**Fig. 6** **&** **7**), and thus cannot draw any conclusions regarding behavioral correlates of the protection by NAC of striatal TH+ fibers. Previous work with this transgenic line indicated that line D SNCA-overexpressing mice exhibited a significant deficit in motor performance compared to their wild-type counterparts [20]. We did not replicate the previous result in this study. The reasons for the increased mean latency to fall across all groups in this work compared to the prior study, despite attempts to follow a standardized protocol, are unclear. This discrepancy highlights the difficulty in quantitative comparisons of behavioral testing results between different laboratories. It will be important to test the protective effect of NAC in other SNCA overexpressing mammalian models of PD that display a more robust behavioral phenotype as well as loss of DA neurons [54]–[57].

NAC is used in humans to treat acetaminophen toxicity [58], and oral supplementation with NAC has been well-tolerated, with promising results for a possible disease-modifying effect in a 6-month controlled trial in patients with Alzheimer's disease [34]. The results presented here demonstrate a neuroprotective effect of oral NAC supplementation, which may be linked to lowering of SNCA levels and mediated in part by increased cytoplasmic sequestration of NFκB, in a chronic degenerative animal model of PD, further strengthening the arguments in favor of consideration of testing NAC as a potential neuroprotective agent for PD.

## Materials and Methods

### Mice

All mice in this study were housed and treated according to the guidelines for the care and use of laboratory animals issued by the Harvard Medical School Institutional Animal Care and Use Committee. A male transgenic mouse overexpressing wild-type SNCA (NACP140) from the PDGFb promoter (line D;[20]) was a kind gift from Pamela McLean (Massachusetts General Hospital). The male was bred with B6D2F1 females (Taconic) to establish the colony. Male offspring expressing the transgene were crossed with wild-type female littermates to maintain one copy of the transgene in subsequent offspring. Male mice were used in all subsequent experiments.

### Genotyping

Genomic DNA was extracted from the ear-punch taken for identification purposes at weaning using the salt-out procedure or 10 minute DNA kit (Crystalgen). PCR primers and amplification protocol were as previously reported [20].

### Water supplementation

Male transgenic or wild-type littermates were randomized to *ad lib* drinking water supplemented with 40 mM NAC or control drinking water with 40 mM alanine at weaning, and remained on supplemented water until sacrifice. Water with NAC or alanine was pH adjusted to pH 7.4 with sodium hydroxide [59] and fresh water was provided thrice weekly. Control drinking water was supplemented with 40 mM alanine, a hydrophillic amino acid, similar in structure to, but lacking the thiol group of cysteine. 40 mM NAC supplemented in this manner has been shown to result in a mean dose of 1 g of NAC per kg body weight per day in C57BL/6 mice, a dose that reduces the accumulation of DNA adducts in mice treated with various carcinogens [60].

### Immunohistochemisty

Cryoprotected brains were cut on a freezing microtome to generate sections of 30 µm thickness that were analyzed for the percentage of striatal area occupied by TH-immunopositive or DAT-immunopositive terminals as previously described [20]. Sections were immunostained for human SNCA with a mouse-on-mouse kit (Vector Labs) according to the manufacturer's protocol using a monoclonal anti-human SNCA antibody with minimal cross-reactivity to mouse SNCA (Cell Sciences Mouse Anti-Human Alpha Synuclein Clone Syn 211 mAb) at a concentration of 1∶500.

### Synuclein Densitometry and Particle Analysis

Sections were imaged using the Axioscop (Zeiss) microscope at 10× magnification using fixed exposure on the SPOT RTkE (Diagnostic Instruments, Inc) imaging system. For densitometric analysis of each section, a region of cortex in the area of M2 was analyzed. The investigator was blinded to genotype and treatment status throughout the process of image examination and acquisition. Gross observation of positively stained slides indicated specific staining that covered most of the section with only the corpus callosum negative for anti-SNCA immunoreactivity. For this reason the corpus callosum was chosen as a control region to control for differences in background staining. Using Adobe Photoshop a series of eight regions of interest (ROI) measuring 50×50 pixels were used. Eight ROIs of 50×50 pixels were chosen since these measurements encompassed the width and length of the corpus callosum at its narrowest part for images taken at ×10 magnification. Integrated density measurements were obtained for both the corpus callosum and cortical region. After subtracting the average integrated density value of the corpus callosum from the integrated density values obtained from the cortical ROIs of that sample and analyzed the results for all four groups using a 2-way ANOVA. As expected, background values from cortex of wild-type animals, which do not express human SNCA, were low. To investigate only the effect of NAC on PDGFb-SNCA mice we performed a two-tailed t-test to compare the difference between this treatment group and PDGFb-SNCA mice treated with alanine. The same strategy was employed to analyze SNCA-immunoreactivity in the striatum. The difference in absolute intensity readings between the cortical and striatal samples demonstrates that the striatal region has much higher non-specific background values for integrated density, as seen in the values obtained for WT animals which do not express human SNCA.

Microscopic observation of PDGFb-SNCA samples stained for human SNCA revealed the obvious presence of highly SNCA-immunopositive cells in both cortex and striatum. The number of these cells per ROI was quantified using the ‘Particle Analysis’ function in ImageJ (NIH, USA). The threshold intensity was set to include those cells that were seen by eye to be positively stained for SNCA and to discount the surrounding background stain. In ImageJ this corresponded to a set threshold of baseline-75 for the cortical region and baseline-24000 for the striatal region. A ROI of 600×600 pixels was set and the ‘Count Particle’ function (with ‘Size (pixel∧2)’ set to 50-infinity and ‘Circularity’ set to 0–1) used to log each cell of the required intensity within this ROI. This method of quantification yielded extremely few false positive cells in wild type animals (average  = 1.5 cells/ROI), therefore the data for PDGFb-SNCA transgenics treated with either alanine or NAC were compared using a two-tailed t-test.

### Tissue preparation and western blot analysis

Animals were overdosed with ketamine/xylazine and intracardiac**-**perfused with ice-cold saline. The brains were removed, placed into a chilled brain matrix and sliced into 1 mm thick coronal sections on ice. The sections were then placed into ice-cold saline. The striatum, and SN were dissected out on a chilled glass dish on ice. Equivalent sized pieces of cortex were also taken from each of the slices containing the striatum and substantia nigra. Tissue samples were homogenized in ice-cold lysis buffer using the TissueLyser LT with 5 mm stainless steel beads (Qiagen). NE-PER Nuclear and Cytoplasmic Extraction Reagents (Thermo Scientific) were used according to the manufacturer's instructions for the extraction of nuclear and cytoplasmic proteins. For whole cell lysate, tissue was homogenized in 50 mM Tris-HCl (pH 7.4), 150 mM NaCl and 1% Triton-X 100 supplemented with protease inhibitors to a final concentration of 1× (Sigma-Aldrich). Protein concentrations were determined by Bicinchoninic Acid (BCA) Assay (Thermo Scientific) according to the manufacturer's instructions. Proteins were separated by SDS-PAGE on a Tris-HCl 4–15% gradient gel (Bio-Rad) according to standard protocols. The membranes were blocked with 5% non-fat milk in either 1× PBS-0.1%Tween or 1× TBS-0.1%Tween. Primary antibodies were diluted in blocking solution in the following manner: rabbit anti-NFκB p65 (A) (sc-109 Santa Cruz) 1∶100; mouse anti-β-actin (C4) (sc-47778 Santa Cruz) 1∶1000; rabbit PDGFb (H-55) (sc-7878 Santa Cruz) 1∶100; rabbit Histone H3 (#9715 Cell Signaling Technology) 1∶1000 and incubated on the membranes with rocking overnight at 4°C. HRP conjugated anti-mouse IgG-HRP or anti-rabbit IgG (Santa Cruz and Cell Signaling Technology) were diluted 1∶1000 in blocking buffer and incubated with the membranes at room temperature for 1 hour. The LumiGOLD ECL Western Blotting Detection Kit (SignaGen) was used according to manufacturer's instructions and the blots were imaged by exposure on Amersham Hyperfilm ECL (GE Healthcare). Band densities were quantified using Adobe Photoshop. Briefly, the image was inverted and the freehand selection tool was used to outline each band. Photoshop automatically computed the integrated density of each band. The integrated density of each band of interest was normalized to the integrated density for the loading control of that band.

### Glutathione assay

Total glutathione was measured by a kinetic spectrophotometric assay based on the reduction of 5′-5′-dithiobis(2-nitrobenzoic acid) (DTNB) measured by change in absorbance at 412 nm as previously described [61]–[63], modified for a 96-well plate format, with each sample measured in duplicate or triplicate. Concentrations of total glutathione (pmol/mg protein) were calculated from a glutathione standard curve run with each experiment. Protein concentration was measured using a BCA Protein Assay Kit (Thermo Scientific) according to the manufacturer's instructions and total cellular glutathione was normalized to the protein content of the sample before calculating the total glutathione content compared to non-NAC treated animals.

### Rotarod Testing

The rotarod is an extensively used test of motor coordination [20], [64], [65]. Mice were tested in the manner outlined in the initial paper describing a locomotor deficit in line D PDGFb-SNCA mice [20]. Briefly, mice were trained on a rotarod for mice (Ugo Basile, Biological Research Apparatus, Varese, Italy) accelerating from 2–40 rpm over a period of 240 seconds for four consecutive days with five repetitions of the task, separated by rest period of 15 minutes, each day. The fifth day constituted the test day and the mice were tested on the same experimental paradigm for a total of seven trials. The average latency to fall was plotted for each genotype for each of those seven test trials.

### Pole Test

The pole test is used to assess motor function in mouse models of basal-ganglia related disorders [66]–[68]. Mice were placed facing upwards at the top of a vertical pole (50 cm in length, 1 cm in diameter) that had been covered with surgical tape to provide a rough surface and capped with a flat plastic disc to prevent the mouse from traversing the top of the pole. The base of the pole was placed in the home cage. The times for the mouse to turn and face downwards (T_TURN_) and for the mouse to descend into the home cage (T_LA_), were recorded with a maximum time of 120 s. Mice were trained on the task for two days with five trials on each day and tested for five trials on the third day. If the mouse fell from the pole or was unable to climb down the pole in any given trial, the longest time from among that animal's previous trials was recorded for the unsuccessful run. This is consistent with previously published methods of analysis of pole test results [69], and an alternative method of excluding such trials did not significantly alter the results in our study.

### Statistical Analyses

Except where otherwise noted, for each of the reported assessments, mean results were compared between groups using a 2-tailed unpaired t-test with a threshold for significance at 0.05.

## Supporting Information

Figure S1 NAC treatment does not affect cellular levels of PDGFb. A. 1 µg of whole-cell protein lysate from 3 alanine- and 3 NAC-treated SNCA-PDGFb striatal samples or B. SN samples were run on a 15-well 4–15% SDS-PAGE gel. After transfer, the membrane was probed with anti- PDGFb antibody (Santa Cruz) and anti-β-actin antibody (Santa Cruz). A single 25 kDa band representing the PDGFb homodimer was seen in most of the striatal samples. The 25 kDa band was seen alongside a 27 kDa band in the SN samples. The 27 kDa band likely represents the PDGFab heterodimer. A single 43 kDa band was seen in for β-actin C. Band quantification of striatal NFκB normalized to β-actin levels. D. Band quantification of SN NFκB normalized to β-actin levels. Only the lower homodimer band was quantified. The experiment was repeated three times. Representative results from a 1 minute exposure are shown. Data were analyzed using a 2-tailed Student's t-test and no statistically significant differences were found for alanine compared to NAC treated striatum or SN.
(0.55 MB TIF)

## References

[1] Thomas B, Beal MF (2007). Parkinson's disease.. Hum Mol Genet.

[2] Schapira AH (2008). Progress in Parkinson's disease.. Eur J Neurol.

[3] Schapira AH (1992). Mitochondrial function in Parkinson's disease. The Royal Kings and Queens Parkinson's Disease Research Group.. Ann Neurol.

[4] Greenamyre JT, Betarbet R, Sherer TB (2003). The rotenone model of Parkinson's disease: genes, environment and mitochondria.. Parkinsonism Relat Disord.

[5] Langston JW (1996). The etiology of Parkinson's disease with emphasis on the MPTP story.. Neurology.

[6] Cassarino DS (1997). Elevated reactive oxygen species and antioxidant enzyme activities in animal and cellular models of Parkinson's disease.. Biochim Biophys Acta.

[7] Kushnareva Y, Murphy AN, Andreyev A (2002). Complex I-mediated reactive oxygen species generation: modulation by cytochrome c and NAD(P)+ oxidation-reduction state.. Biochem J.

[8] Sherer TB (2002). An In Vitro Model of Parkinson's Disease: Linking Mitochondrial Impairment to Altered a-Synuclein Metabolism and Oxidative Damage.. J Neurosci.

[9] Jenner P (2003). Oxidative stress in Parkinson's disease.. Ann Neurol.

[10] Perry TL, Godin DV, Hansen S (1982). Parkinson's disease: a disorder due to nigral glutathione deficiency?. Neurosci Lett.

[11] Dexter DT (1994). Increased levels of lipid hydroperoxides in the parkinsonian substantia nigra: an HPLC and ESR study.. Mov Disord.

[12] Rochet JC (2004). Interactions among alpha-synuclein, dopamine, and biomembranes: some clues for understanding neurodegeneration in Parkinson's disease.. J Mol Neurosci.

[13] Xu J (2002). Dopamine-dependent neurotoxicity of alpha-synuclein: a mechanism for selective neurodegeneration in Parkinson disease.. Nat Med.

[14] Duda JE (2000). Widespread nitration of pathological inclusions in neurodegenerative synucleinopathies.. Am J Pathol.

[15] Souza JM (2000). Dityrosine cross-linking promotes formation of stable alpha -synuclein polymers. Implication of nitrative and oxidative stress in the pathogenesis of neurodegenerative synucleinopathies.. J Biol Chem.

[16] Giasson BI (2000). Oxidative damage linked to neurodegeneration by selective alpha- synuclein nitration in synucleinopathy lesions.. Science.

[17] Hashimoto M (1999). Oxidative stress induces amyloid-like aggregate formation of NACP/alpha-synuclein in vitro.. Neuroreport.

[18] Paxinou E (2001). Induction of alpha-synuclein aggregation by intracellular nitrative insult.. J Neurosci.

[19] Conway KA (2001). Kinetic Stabilization of the alpha -Synuclein Protofibril by a Dopamine- alpha -Synuclein Adduct.. Science.

[20] Masliah E (2000). Dopaminergic loss and inclusion body formation in alpha-synuclein mice: implications for neurodegenerative disorders.. Science.

[21] Ibanez P (2004). Causal relation between alpha-synuclein gene duplication and familial Parkinson's disease.. Lancet.

[22] Chartier-Harlin MC (2004). Alpha-synuclein locus duplication as a cause of familial Parkinson's disease.. Lancet.

[23] Singleton AB (2003). alpha-Synuclein locus triplication causes Parkinson's disease.. Science.

[24] Dringen R, Hamprecht B (1999). N-acetylcysteine, but not methionine or 2-oxothiazolidine-4-carboxylate, serves as cysteine donor for the synthesis of glutathione in cultured neurons derived from embryonal rat brain.. Neurosci Lett.

[25] Zafarullah M (2003). Molecular mechanisms of N-acetylcysteine actions.. Cellular and Molecular Life Sciences (CMLS).

[26] Reliene R, Fischer E, Schiestl RH (2004). Effect of N-acetyl cysteine on oxidative DNA damage and the frequency of DNA deletions in atm-deficient mice.. Cancer Res.

[27] Pocernich CB, La Fontaine M, Butterfield DA (2000). In-vivo glutathione elevation protects against hydroxyl free radical- induced protein oxidation in rat brain.. Neurochem Int.

[28] Banaclocha MM (2000). N-acetylcysteine elicited increase in complex I activity in synaptic mitochondria from aged mice: implications for treatment of Parkinson's disease.. Brain Res.

[29] Banaclocha MM (1997). N-acetylcysteine protects against age-related increase in oxidized proteins in mouse synaptic mitochondria.. Brain Res.

[30] Vina J (1983). Effects of cysteine and N-acetyl cysteine on GSH content of brain of adult rats.. Experientia.

[31] Sheffner AL (1966). Metabolic studies with acetylcysteine.. Biochem Pharmacol.

[32] Perry TL (1985). Partial protection from the dopaminergic neurotoxin N-methyl-4-phenyl- 1,2,3,6-tetrahydropyridine by four different antioxidants in the mouse.. Neurosci Lett.

[33] Sharma A (2007). Attenuation of 1-methyl-4-phenyl-1, 2,3,6-tetrahydropyridine induced nigrostriatal toxicity in mice by N-acetyl cysteine.. Cell Mol Biol (Noisy-le-grand).

[34] Adair JC, Knoefel JE, Morgan N (2001). Controlled trial of N-acetylcysteine for patients with probable Alzheimer's disease.. Neurology.

[35] Banaclocha MM (2001). Therapeutic potential of N-acetylcysteine in age-related mitochondrial neurodegenerative diseases.. Med Hypotheses.

[36] Martinez M (1999). Hypothesis: can N-acetylcysteine be beneficial in Parkinson's disease?. Life Sci.

[37] Ravina BM (2003). Neuroprotective agents for clinical trials in Parkinson's disease: a systematic assessment.. Neurology.

[38] Aoyama K (2006). Neuronal glutathione deficiency and age-dependent neurodegeneration in the EAAC1 deficient mouse.. Nat Neurosci.

[39] Liu L (2010). Ghrelin prevents 1-methyl-4-phenylpyridinium ion-induced cytotoxicity through antioxidation and NF-kappaB modulation in MES23.5 cells.. Exp Neurol.

[40] Sha D, Chin LS, Li L (2010). Phosphorylation of parkin by Parkinson disease-linked kinase PINK1 activates parkin E3 ligase function and NF-kappaB signaling.. Hum Mol Genet.

[41] Aoki E (2009). Role of nuclear transcription factor kappa B (NF-kappaB) for MPTP (1-methyl-4-phenyl-1,2,3,6-tetrahyropyridine)-induced apoptosis in nigral neurons of mice.. Exp Mol Pathol.

[42] Camandola S, Mattson MP (2007). NF-kB as a therapeutic target in neurodegenerative diseases.. Expert Opinion on Therapeutic Targets.

[43] Khachigian LM (1995). Nuclear factor-kappa B interacts functionally with the platelet-derived growth factor B-chain shear-stress response element in vascular endothelial cells exposed to fluid shear stress.. J Clin Invest.

[44] Shi J (2004). Roles of NF-kappaB and SP-1 in oxidative stress-mediated induction of platelet-derived growth factor-B by TNFalpha in human endothelial cells.. J Cardiovasc Pharmacol.

[45] Chesselet MF (2008). Strengths and limitations of genetic mouse models of Parkinson's disease.. Parkinsonism Relat Disord.

[46] Hashimoto M, Rockenstein E, Masliah E (2003). Transgenic models of alpha-synuclein pathology: past, present, and future.. Ann N Y Acad Sci.

[47] Hardy J (2006). Genetics of Parkinson's disease and parkinsonism.. Annals of Neurology.

[48] Gwinn-Hardy K (2002). Genetics of parkinsonism.. Mov Disord.

[49] Adams JD, Chang ML, Klaidman L (2001). Parkinson's disease–redox mechanisms.. Curr Med Chem.

[50] Quilty MC (2006). Alpha-synuclein is upregulated in neurones in response to chronic oxidative stress and is associated with neuroprotection.. Exp Neurol.

[51] Ghosh A (2007). Selective inhibition of NF-kappaB activation prevents dopaminergic neuronal loss in a mouse model of Parkinson's disease.. Proc Natl Acad Sci U S A.

[52] Lee H-J (2010). Direct Transfer of a-Synuclein from Neuron to Astroglia Causes Inflammatory Responses in Synucleinopathies.. Journal of Biological Chemistry.

[53] Gu X-L (2010). Astrocytic expression of Parkinson's disease-related A53T alpha-synuclein causes neurodegeneration in mice.. Molecular Brain.

[54] Kirik D (2002). Parkinson-like neurodegeneration induced by targeted overexpression of alpha-synuclein in the nigrostriatal system.. J Neurosci.

[55] Kirik D (2003). Nigrostriatal alpha-synucleinopathy induced by viral vector-mediated overexpression of human alpha-synuclein: a new primate model of Parkinson's disease.. Proc Natl Acad Sci U S A.

[56] Richfield EK (2002). Behavioral and neurochemical effects of wild-type and mutated human alpha-synuclein in transgenic mice.. Exp Neurol.

[57] Thiruchelvam MJ (2004). Risk factors for dopaminergic neuron loss in human alpha-synuclein transgenic mice.. Eur J Neurosci.

[58] Zed PJ, Krenzelok EP (1999). Treatment of acetaminophen overdose.. Am J Health Syst Pharm.

[59] Andreassen OA (2000). N-acetyl-L-cysteine improves survival and preserves motor performance in an animal model of familial amyotrophic lateral sclerosis.. Neuroreport.

[60] Balansky R (1996). Induction by carcinogens and chemoprevention by N-acetylcysteine of adducts to mitochondrial DNA in rat organs.. Cancer Res.

[61] Tietze F (1969). Enzymic method for quantitative determination of nanogram amounts of total and oxidized glutathione: applications to mammalian blood and other tissues.. Anal Biochem.

[62] Griffith OW (1980). Determination of glutathione and glutathione disulfide using glutathione reductase and 2-vinylpyridine.. Anal Biochem.

[63] Vornov JJ, Park J, Thomas AG (1998). Regional vulnerability to endogenous and exogenous oxidative stress in organotypic hippocampal culture.. Exp Neurol.

[64] Jones BJ, Roberts DJ (1968). The quantitative measurement of motor inco-ordination in naive mice using an accelerating rotarod.. J Pharm Pharmacol.

[65] Tillerson JL (2002). Detection of behavioral impairments correlated to neurochemical deficits in mice treated with moderate doses of 1-methyl-4-phenyl-1,2,3,6-tetrahydropyridine.. Exp Neurol.

[66] Ogawa N (1985). A simple quantitative bradykinesia test in MPTP-treated mice.. Res Commun Chem Pathol Pharmacol.

[67] Matsuura K (1997). Pole test is a useful method for evaluating the mouse movement disorder caused by striatal dopamine depletion.. Journal of Neuroscience Methods.

[68] Fleming SM (2004). Early and progressive sensorimotor anomalies in mice overexpressing wild-type human alpha-synuclein.. J Neurosci.

[69] Rommelfanger KS (2007). Norepinephrine loss produces more profound motor deficits than MPTP treatment in mice.. Proceedings of the National Academy of Sciences.

