# Supplementary figures and images for: Oral N-Acetyl-Cysteine Attenuates Loss of Dopaminergic Terminals in α-Synuclein Overexpressing Mice
Source: PLoS One. 2010 Aug 23;5(8):e12333. doi: 10.1371/journal.pone.0012333 (PMC2925900; doi:10.1371/journal.pone.0012333)

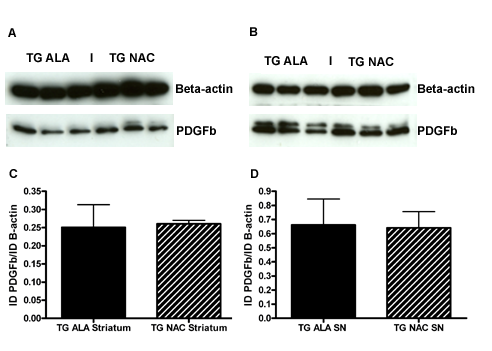

Supplement: Figure S1 — NAC treatment does not affect cellular levels of PDGFb. A. 1 µg of whole-cell protein lysate from 3 alanine- and 3 NAC-treated SNCA-PDGFb striatal samples or B. SN samples were run on a 15-well 4–15% SDS-PAGE gel. After transfer, the membrane was probed with anti- PDGFb antibody (Santa Cruz) and anti-β-actin antibody (Santa Cruz). A single 25 kDa band representing the PDGFb homodimer was seen in most of the striatal samples. The 25 kDa band was seen alongside a 27 kDa band in the SN samples. The 27 kDa band likely represents the PDGFab heterodimer. A single 43 kDa band was seen in for β-actin C. Band quantification of striatal NFκB normalized to β-actin levels. D. Band quantification of SN NFκB normalized to β-actin levels. Only the lower homodimer band was quantified. The experiment was repeated three times. Representative results from a 1 minute exposure are shown. Data were analyzed using a 2-tailed Student's t-test and no statistically significant differences were found for alanine compared to NAC treated striatum or SN. (0.55 MB TIF) [file pone.0012333.s001.tif]
